# Case Report: Neo-homozygous nonsense mutation in NLRP5 associated with early embryonic arrest in two sisters from a Chinese family

**DOI:** 10.3389/frph.2026.1767934

**Published:** 2026-02-17

**Authors:** Qin Xu, Yumei Deng, Jingjing Huo, Yuanlong Yan, Yangjia Zhang, Yaxian Ma, Li Zhuan

**Affiliations:** 1 Department of Reproductive Medicine, The First People’s Hospital of Yunnan Province, Kunming, Yunnan, China; 2 Department of Medical Genetics, The First People’s Hospital of Yunnan Province, Kunming, Yunnan, China; 3 Department of Reproductive Medicine, Yunnan Maternal and Child Health Hospital, Kunming, Yunnan, China

**Keywords:** assisted reproductive technology, early embryonic arrest, NLRP5, novel mutation, terminator codon

## Abstract

Early embryonic arrest (EEA) can result in repeated failures of assisted reproductive technology, with genetic variation being the primary cause. The maternal protein nucleotide-binding oligomerization domain-like receptor protein 5 (NLRP5) plays a role in oocyte maturation and embryonic development before the blastocyst stage. Mutations in the NLRP5 gene can lead to various reproductive outcomes, including oocyte maturation disorder, fertilization failure, and EEA. We discovered a new homozygous nonsense mutation (c.779G > A; p.Trp260*) in NLRP5 in two sisters from a Chinese family. This clinically presented as halted embryonic development at the 2–7 cell stage. The parents and brother were heterozygous carriers and exhibited normal fertility, indicating that the pathogenic gene was inherited in an autosomal recessive manner. Analyses revealed significantly decreased expression of NLRP5 at the 3' end of the mRNA and the C-terminal of the protein *in vitro* (*p* < 0.05). This suggests that NLRP5 protein dysfunction is the primary cause of EEA in this case. Additionally, the expression levels are inconsistent with those of previous studies, indicating that different mutation sites lead to variations in NLRP5 protein expression and distinct pathogenic mechanisms. Our finding expands the spectrum of pathogenic variants in EEA caused by the NLRP5 gene.

## 1 Introduction

Infertility, affecting up to 25% of women of reproductive age, is increasing annually, posing a significant social and public health challenge. Human-assisted reproductive technology (ART) is an important approach to managing infertility. Early embryonic arrest (EEA) refers to the arrest of growth and development of the embryo from fertilization to the blastocyst stage. Studies have shown that more than 40% of ART patients cannot transfer embryos due to EEA, which remains one of the leading causes of repeated ART failure. EEA has a high incidence in patients with primary unexplained infertility, mainly due to genetic and environmental factors (1).

Cytoplasmic lattices are important structures for oocytes to store proteins necessary for EEA. These lattices are composed of peptidyl arginine deaminase 6 and subcortical maternal complex (SCMC) (1). Among these, SCMC is a polyprotein complex enriched in oocytes and the subcortical region of preimplantation embryos, crucial for the development after the first meiosis of fertilized eggs (2). Additionally, related gene variants have been implicated in EEA (3, 4).

Nucleotide-binding oligomerization domain-like receptor protein 5 (NLRP5), also known as MATER, is the core component of SCMC. It comprises the following three distinct domains: the N-terminal Pyrin domain, the central nucleotide-binding oligomerization domain (NACHT), and the C-terminal leucine-rich repeat (LRR) domain. The Pyrin domain facilitates binding with adaptor proteins and is essential for the stability of SCMC; the NACHT domain binds ATP; the LRR domain plays a role in protein-protein interactions (5–8). Studies have shown that different NLRP5 gene variants are associated with adverse reproductive outcomes, including oocyte maturation disorder, fertilization failure, and EEA. However, the specific mechanisms remain unclear (5).

In this study, we found a new homozygous nonsense mutation of NLRP5 (c.779G > A; P.Trp260*) in two sisters from a consanguineous Chinese family. This mutation interferes with the expression and function of the NLRP5 protein, leading to EEA and infertility.

## 2 Materials and methods

### 2.1 Study participant and informed consent

Two sisters and one brother in a family were treated for infertility at the Department of Reproductive Medicine of the First People's Hospital of Yunnan Province, Kunming, China. Their parents are first cousins.

The patients provided written informed consent for publication.

This study involves human participants and was approved by the hospital Ethics Committee (number:2025-GN001).

### 2.2 Controlled ovarian hyperstimulation

Elder sister: Diagnosed with “primary infertility for 5 years and bilateral fallopian tube obstruction,” and *in vitro* fertilization (IVF) was performed over two cycles.

The first cycle:

- The ovarian stimulation protocol: Gonadotrophin-releasing hormone agonist, initiated with daily administration of 150 IU of Gonal-F, with 75 IU of human menopausal gonadotrophin added on the 6th day.
- Trigger: When two or three follicles reached a diameter of more than 18 mm, recombinant human chorionic gonadotropin (rHCG, Ovidrel, 250 µg, subcutaneous injection) was administrated with a duration of action of 37 h.

The second cycle:

- Superovulation was induced using an antagonist regimen, starting with daily administration of 150 IU of recombinant follitropin beta injection for 9 days.
- Trigger: Ovulation was triggered with rHCG (250 µg, subcutaneous injection) with a duration of action of 37 h.

Younger sister: Diagnosed with “primary infertility for 4 years and bilateral fallopian tube obstruction,” and IVF was performed for one cycle.

- Superovulation was induced using an antagonist regimen, starting with daily administration of 100 IU of recombinant follitropin beta injection for 12 days.
- Trigger: Ovulation was triggered with rHCG (250 µg, subcutaneous injection) with a duration of action of 37 h.

### 2.3 Insemination and embryo culture

On the day of oocyte retrieval, semen samples were collected concurrently and processed through standard semen analysis and optimization. For insemination, a sperm-to-oocyte ratio of 30,000:2 was used. The oocytes were then co-incubated with the prepared sperm. At 4–6 h post-insemination, denudation was performed to assess for the presence of the second polar body. Normal fertilization was evaluated 16–18 h after insemination and confirmed by the observation of two pronuclei. Embryo development was monitored daily at a fixed time point (8:00 a.m.) from day 2 to day 3 after insemination, with all morphological assessments recorded accordingly.

### 2.4 Family gene analysis

Blood samples were collected from the two sisters and their parents, and a genomic library based on the subjects’ genomic DNA was constructed for each patient. Relevant target gene fragments were captured and enriched. The enriched target gene fragments were sequenced using the MGISEQ-2000 sequencing platform, with an average sequencing depth of ≥180X in the target area. The proportion of sites with an average depth >20X was more than 95%. Using the human genome reference sequence hg19/GRCh37, whole-exome sequencing was performed on the family. The genome aggregation database (gnomAD), the 1000 Genomes, the online Mendelian inheritance in man, the exome aggregation consortium (ExAC), ClinVar, and the human gene mutation database were employed as references. Variant annotation complied with the guidelines of the American College of Medical Genetics (ACMG). Raw sequencing reads were filtered, and the selected variants were subjected to validation using Sanger sequencing. In addition to both sisters and their parents, DNA samples of the brother were also subjected to Sanger sequencing.

### 2.5 Plasmid construction and quantitative real-time PCR (qPCR)

Wild-type and mutant NLRP5 cDNA plasmids were constructed and supplied by Yunzhou Biotechnology Co., LTD. (China). The constructs were verified via Sanger sequencing. RNA was extracted using TRizol, and cDNA was obtained via the SweScript-First-strand-cDNA-synthesis-kit for reverse transcription. qPCR experiments were performed on the synthesized cDNA with primers, including:

NLRP5-3' primers:

- Forward (F): CCACAGGTCCTACTTGCTCTA
- Reverse (R): TGAGCGATTTGTCTCCTTCCA

NLRP5-5' primers:

- Forward (F): CCACGTGCTCTTGTCACTCTT
- Reverse (R): TAGAGACACCATTGCAGCCC

Parameter H-GAPDH (reference gene)

- Forward (F): ATGGGCAGCCGTTAGGAAAG
- Reverse (R): AGGAAAAGCATCACCCGGAG.

The mRNA levels of each group were quantitatively analyzed using the 2^−*ΔΔ*CT^ method. The experiments were performed with 3 independent biological replicates.

### 2.6 Western blotting

Forty-eight hours after transfection with the plasmid, total protein was extracted from HEK293 T cells through radioimmunoprecipitation lysis (Servicebio, China). Protein concentrations were determined using the BCA kit (Biyuntian Company, China). The proteins of each group were separated using 10% sodium dodecyl sulfate–polyacrylamide gel electrophoresis (Solarbio, China) and transferred onto polyvinylidene difluoride membranes (Millipore, USA) via the wet rotation method. The membranes were blocked with 5% bovine serum albumin (Solarbio, China) and incubated overnight with NLRP5 (1:1,000 dilution, Invitrogen, USA, raised against a C-terminal epitope corresponding to amino acids 650-950 of human NLRP5) and *β*-Actin (1:25,000 dilution, Proteintech, China) primary antibodies at 4 °C. This was followed by rewarming incubation with HRP-labeled goat anti-rabbit IgG (1:250 dilution, Invitrogen, USA) and HRP-labeled goat anti-mouse IgG (1:500, Servicebio, China). The experiments were performed with 3 independent biological replicates.The ImageJ and Prism softwares were used for gray value and bar chart statistics, respectively.

## 3 Results

### 3.1 Clinical characteristics

The clinical data of the two sisters, including infertility duration, age, height, weight, body mass index, baseline sex hormones, anti-Müllerian hormone levels, antral follicle count, and chromosome karyotype, are presented in Table 1. Both sisters' husbands had a 46, XY karyotype and normal sperm parameters. Semen analysis results of the brother revealed a semen volume of 0.4 mL, a sperm density of 2.26 × 10^6^/mL, a forward motility sperm rate of 11.73%, and a deformity rate of 28.28%. The karyotype was normal. Semen analysis was conducted based on the World Health Organization 2010 guidelines, 5th edition (9).

**Table 1:** The basic clinical characteristics of both sisters.

Characteristics	Sister 1	Sister 2
Infertility years	5	4
Infertility nature	Primary	Primary
Age (years)	34	30
Height (cm)	158	155
Weight (kg)	55	43
BMI (kg/m^2^)	22.03	17.90
FSH (mIU/mL)	5.27	6.83
LH (mIU/mL)	1.23	4.73
E_2_ (pmol/L)	45.00	52.00
AMH (ng/mL)	1.43	6.0
AFC	10	16
Chromosome karyotype	46, XX	46, XX

BMI, body mass index; FSH, follicle-stimulating hormone; LH, luteinizing hormone; E_2_, estradiol; P, progesterone; AMH, anti-Müllerian hormone; AFC, antral follicle count.

The elder sister received two cycles of IVF, with a total of 15 eggs obtained, and embryo development stagnated in the 2–4 cell stage. The younger sister received one cycle of IVF, with a total of 16 eggs obtained, and halted embryonic development in the 2–7 cell stage (Supplementary Table S1). She did not achieve pregnancy following the transfer of a 7-cell embryo. While the brother was preparing to undergo intracytoplasmic sperm injection, his wife conceived naturally and delivered a healthy baby boy.

The two sisters were clinically followed for two years after genetic diagnosis. During this period, neither sister achieved a successful pregnancy. We repeatedly counseled them regarding alternative reproductive options, including the use of donor oocytes. Both patients were fully informed and indicated that they were in the process of considering and arranging for such treatment. No adverse or unanticipated events occurred throughout the follow-up period.

### 3.2 Identification of a novel mutation in NLRP5

**Figure 1:**
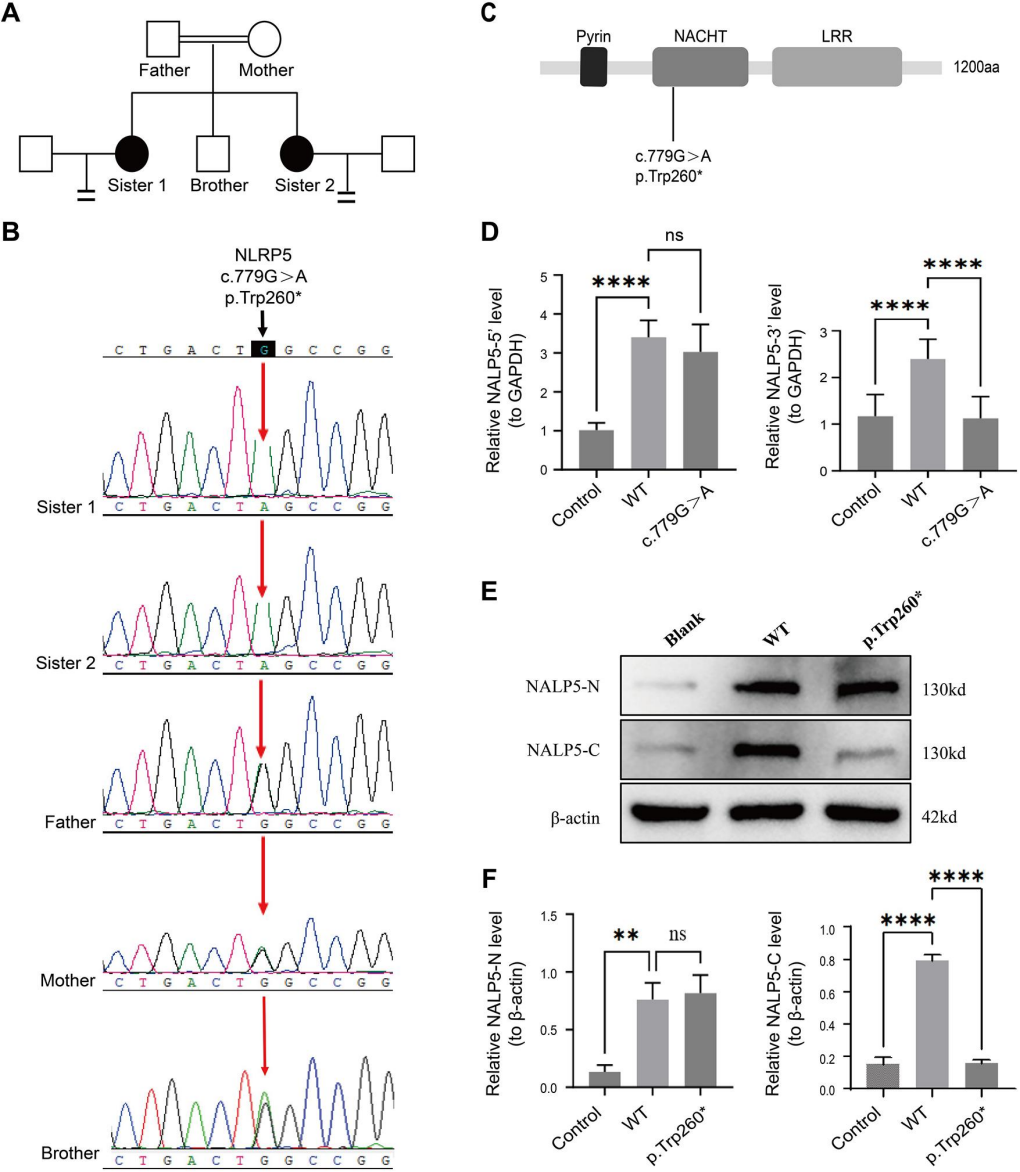
Identification of a neo-homozygous nonsense mutation of NLRP5 in two sisters in a Chinese family with early embryonic arrest. **(A)** Pedigree of the family. Squares denote male family members, circles denote female members, black solid circles denote the patient, the double line represents consanguinity, and equal signs indicate infertility. **(B)** Sanger sequencing electropherograms of the family. **(C)** The structure and mutant residue of NLRP5. **(D)** qPCR analysis of NLRP5-3’ and NLRP5-5’ in HEK293T cells with wild-type (WT) and p.Trp260* plasmids. **(E)** Western blotting of NLRP5-N and NLRP5-C in HEK293T cells with WT and p.Trp260* plasmids. **(F)** Quantitative analysis of western blotting. ns: not significant. The qPCR and Western blotting were performed with 3 independent biological replicates. ns: not significant *: *p* < 0.05. NLRP5, nucleotide-binding oligomerization domain-like receptor protein 5; qPCR, quantitative real-time PCR.

The homozygous mutation (c.779G > A) in exon 7 of the NLRP5 gene (NM_153447.4) in both sisters, which may be associated with EEA, was identified via whole-exome gene sequencing, resulting in the conversion of the 260th tryptophan to the termination code (p.Trp260*). Both parents were found to be heterozygous carriers of the mutation. Sanger sequencing also confirmed homozygous mutations in both sisters and heterozygous mutations in their parents and brother (Figures 1A,B). This variant was located in the conserved NACHT domain of the NLRP family (Figure 1C) and was not present in Thousand Genomes, ESP6500, ExAC, or GnomAD databases. According to the ACMG guidelines, this variant was suspected to be pathogenic. qPCR and Western blotting were performed to assess the impact of the mutation on NLRP5 expression. the data indicate that the mutation led to a significant decrease in the signal from the 3' region of NLRP5 mRNA and a marked reduction in overall NLRP5 protein levels detectable by our C-terminal antibody, compared with the wild type (*p* < 0.05) (Figures 1D–F).

## 4 Discussion

NLRP5 is expressed in ovaries, MII eggs, two-cell embryos, and granulosa cells of buffalo and significantly decreases after the 8-cell stage. In contrast, it is hardly detected in blastocysts, indicating that NLRP5 is a maternal protein associated with reproduction and mainly plays a role in the development of eggs and embryos before blastocyst formation (10). Mutations in the NLRP5 gene can lead to varying reproductive outcomes. For example, c.1286_1289del (p.V429Efs*30) variation can lead to oocyte maturation dysfunction, which may be mediated by the nonsense-mediated mRNA decay signaling pathway, resulting in rapid NLRP5mRNA degradation to protein failure. The loss of protein function may also be caused by the deletion of NACHT fragments (11). The c.1598G > C and c.1919T > G (p.L640R) variants are associated with fertilization failure (12), whereas the c.1093G > A (p.Asp365Asn) variant is likely related to recurrent molar pregnancies (13).

Several studies have reported that NLRP5 variants are associated with EEA. For example, patients with c.971T > A, c.3341T > C, c.1575_1576 delAG, c.1830_1831delGT, c.1202C > T, and c.2378T > G variants exhibit higher rates of embryo fragmentation (40%–80%) than those with the 2-cell to 4-cell stage (14). Additionally, c.1061C > T (p.Pro354Leu) and c.1903C > T (p.Arg635Cys) mutations usually result in embryos stagnating at the 3–7 cell stage, with only a few developing to the 8-cell stage. Subsequent blastocyst culture and post-transplant pregnancy are frequently unsuccessful (15, 16). Variants, such as the c.292C > T(p.Gln98*), c.866G > A(p.Gly289Glu), c.2081C > T(p.Thr694Ile), and c.3320C > T(p.Thr1107Ile) usually lead to EEA by reducing NLRP5 protein expression in eggs and embryos (17). However, these known variants do not fully account for all cases of EEA. It is important to note that the differential diagnosis of EEA also includes mutations in other maternal-effect genes, chromosomal abnormalities, and metabolic factors.

We found that two sisters in a family had a novel homozygous nonsense mutation in NLRP5 (c.779G > A; p.Trp260*), which resulted in embryonic development stagnating at the 2–7 cell stage. One 7-cell embryo was not pregnant after transfer, which was consistent with previous studies. In this family, both parents and brother were heterozygous and had normal reproductive function, indicating that the pathogenic gene was inherited in an autosomal recessive manner. NLRP5 contains 1,200 amino acids and comprises the following three functional domains: the N-terminal Pyrin, middle NACHT, and C-terminal LRR domains. The NACHT domain contains an evolutionarily conserved NTPase domain in the NLRP protein family. Although ATP or GTP binds to the NTPase domain, the energy-producing hydrolysis of NTP can subsequently induce conformational changes in adjacent regions of the protein (18). Xu et al. found that mutations in the NACHT domain reduced the expression of NLRP5 *in vitro*, which is consistent with that of the wild type and may lead to EEA by interfering with molecular interactions and disrupting NTPase activity (16). In this study, the mutation site was also located in the NACHT domain; however, the mutation caused the termination code to appear at the 260th amino acid, resulting in significantly reduced expression of NLRP5 protein *in vitro*. This indicates that both the NACHT and LRR domains lost function, further contributing to EEA. The expression of NLRP5 *in vitro* in our case was consistent with that found by Mu et al. (17), but inconsistent with that reported by Xu et al. (16), indicating that different mutation sites lead to different expressions of NLRP5 protein. Therefore, the pathogenic mechanisms may also be different.

This study had some limitations. First, the patient's oocytes and embryos were not obtained, and the expression of NLRP5 in patients was not directly evaluated. Second, the functional validation in this study was performed in HEK293 T cells, a non-reproductive cell line. Although this approach provided initial evidence for the variant's impact on mRNA and protein expression, it may not fully recapitulate NLRP5's physiological role and interactions within oocytes and early embryos. While studies in mouse models have shown that NLRP5 is involved in key processes such as mitochondrial activation, endoplasmic reticulum distribution, and calcium homeostasis (5), the scarcity of human oocytes and embryos limits our ability to fully elucidate the specific molecular mechanisms by which NLRP5 dysfunction leads to EEA. Future studies using more physiologically relevant models—such as patient-derived induced pluripotent stem cells (iPSCs) differentiated toward the germline lineage or functional rescue experiments in mouse oocytes—would be valuable to confirm the pathogenic mechanism and further understand its role in human early development. Otherwise, while the clinical and genetic findings are described in detail, direct patient-reported perspectives or structured interviews were not included in this study,the future studies could incorporate patient voice to enrich the psychosocial and ethical dimensions of such cases.

## 5 Conclusion

We identified a new homozygous nonsense mutation of NLRP5 (c.779G > A; p.Trp260*) in two sisters from a Chinese family with clinical manifestations of EEA and infertility. This finding expands the known spectrum of clinically pathogenic variants in the NLRP5 gene.

## Data Availability

The original contributions presented in the study are included in the article/Supplementary Material, further inquiries can be directed to the corresponding author/s.
