# Supplementary material for: Case Report: Neo-homozygous nonsense mutation in NLRP5 associated with early embryonic arrest in two sisters from a Chinese family
Source: Front Reprod Health. 2026 Feb 17;8:1767934. doi: 10.3389/frph.2026.1767934 (PMC12953539; doi:10.3389/frph.2026.1767934)
Supplement: Supplementary file 1 [file Table1.docx]

Supplementary Material

**Supplemental Table 1:** Embryo culture of the two sisters

|  |  |  | D1  Cleaved to 2-cell | D2  2-cell cleaved to 4-cell | D3  4-cell cleaved to 8-cell | Outcomes |
| --- | --- | --- | --- | --- | --- | --- |
| Sister 1 | **1^st^ IVF** | Embryo 1 | MII | MII | / | embryonic arrest |
|  |  | Embryo 2 | MII | 2cell | 2cell | embryonic arrest |
|  |  | Embryo 3 | 2PB | / | / | / |
|  |  | Embryo 4 | MII | 2cell | 2cell | embryonic arrest |
|  |  | Embryo 5 | MI | / | / | / |
|  |  | Embryo 6 | 1PN | 3cell | 3cell | embryonic arrest |
|  |  | Embryo 7 | MI | / | / | / |
|  |  | Embryo 8 | MII | MII | / | / |
|  |  | Embryo 9 | MII | MII | / | / |
|  |  | Embryo 1 | MII | 2cell | 2cell | embryonic arrest |
|  |  | Embryo 2 | MII | 2cell | 2cell | embryonic arrest |
|  |  | Embryo 3 | MI | / | / | / |
|  | **2^nd^ IVF** | Embryo 4 | MII | 2cell | 2cell | embryonic arrest |
|  |  | Embryo 5 | MII | 4cell | 4cell | embryonic arrest |
|  |  | Embryo 6 | MI | / | / | / |
| Sister 2 | **1^st^ IVF** | Embryo 1 | 1PN | 1PN | / | / |
|  |  | Embryo 2 | MII | MII | / | / |
|  |  | Embryo 3 | MII | 3cell | 3cell | embryonic arrest |
|  |  | Embryo 4 | MII | MII | / | / |
|  |  | Embryo 5 | 2PN | 2PN | / | / |
|  |  | Embryo 6 | 1PN | 2cell | 2cell | embryonic arrest |
|  |  | Embryo 7 | MII | MII | / | / |
|  |  | Embryo 8 | 1PN | 5cell | 5cell | embryonic arrest |
|  |  | Embryo 9 | 2PN | 2cell | 2cell | embryonic arrest |
|  |  | Embryo 10 | 2PN | 2PN | / | / |
|  |  | Embryo 11 | MII | 2cell | 2cell | embryonic arrest |
|  |  | Embryo 12 | 2PN | 2cell | 2cell | embryonic arrest |
|  |  | Embryo 13 | 1PN | 2cell | 2cell | embryonic arrest |
|  |  | Embryo 14 | MII | 2cell | 2cell | embryonic arrest |
|  |  | Embryo 15 | 2PN | 5cell | 7cell | Grade III 7-cell stage |
|  |  | Embryo 16 | 2PN | 2pn | / | / |

**Abbreviations:** D1: the first day; D2: the second day; D3: the third day; IVF: *in vitro* fertilization; MII: meiosis II; MI: metaphase I; PB: polar body; PN: pronucleus.
